# Reevaluating Melatonin’s Efficacy in Alzheimer’s Disease: A 20th‐Anniversary Updated Literature Review of Sleep Disturbances and Treatment Trials

**DOI:** 10.1002/alz.095541

**Published:** 2025-01-09

**Authors:** Alisha Malhotra, Vrushabh Daga, Cliff Singer

**Affiliations:** ^1^ University of New England, Biddeford, ME USA; ^2^ Northern Light Health, Bangor, ME USA

## Abstract

Sleep disturbances in Alzheimer’s disease (AD) are stressful for patients and families. This review provides an update on the efficacy of melatonin and other interventions for nighttime sleep disturbances in AD patients, building on the original Alzheimer’s disease Cooperative Study trial, the first large trial for sleep disturbance in AD. The initial multicenter, randomized, placebo‐controlled study investigated the effects of two melatonin formulations – 2.5 mg sustained‐release (ML2.5SR) and 10 mg immediate‐release (ML10) –compared to placebo (PLA) in 157 participants. The primary outcomes, measured via wrist‐actigraphy, indicated no significant changes; however, secondary outcomes suggested improvement in sleep quality with ML2.5SR. This suggests the efficacy of melatonin in AD may be related to time and exposure rather than dosage, as sustained‐release formulations showed a greater trend for efficacy than immediate.

Melatonin’s antioxidant properties have been shown to be neuroprotective, balancing Aβ production and clearance, but have been equivocal in reducing sleep disturbances. Recent research has demonstrated actigraphy to be an imperfect method for measuring sleep and wakefulness, misclassifying asleep time as awake in people with AD, and overestimating sleep in others. Combined with the subjective nature and poor compliance with sleep diaries in the ADCS study, methodological questions as to how to do large studies of sleep in people with AD arose. Updated studies have looked at melatonin in comparison to interventions like bright‐light therapy and other drugs. Circadian rhythm disturbances are common in AD, and different therapies have been trialed to improve sleep onset, duration, and quality. There has also been updated information regarding the efficacy of different measure to accurately record sleep including EEG and polysomnography.

We analyzed studies in the 20 years since the ADCS trial was published. Our findings, while mixed, suggest that melatonin may be more effective improving sleep outcomes in AD, than reported in the ADCS paper‐ particularly when used in conjunction with other interventions. Suggestions for further study include finding a new way to objectively gather sleep data in a large trial so that another multicenter trial looking several sustained release formulations and other interventions may be attempted.

